# 5-Cyclo­hexyl-3-methyl­sulfinyl-2-phenyl-1-benzofuran

**DOI:** 10.1107/S1600536810054358

**Published:** 2011-01-08

**Authors:** Hong Dae Choi, Pil Ja Seo, Byeng Wha Son, Uk Lee

**Affiliations:** a Department of Chemistry, Dongeui University, San 24 Kaya-dong Busanjin-gu, Busan 614-714, Republic of Korea; b Department of Chemistry, Pukyong National University, 599-1 Daeyeon 3-dong, Nam-gu, Busan 608-737, Republic of Korea

## Abstract

In the title compound, C_21_H_22_O_2_S, the cyclo­hexyl ring adopts a chair conformation while the phenyl ring makes a dihedral angle of 33.38 (5)° with the mean plane of the benzofuran fragment. In the crystal, mol­ecules are linked through weak inter­molecular C—H⋯O and C—H⋯π inter­actions.

## Related literature

For the biological activity of benzofuran compounds, see: Aslam *et al.* (2006 ▶); Galal *et al.* (2009 ▶); Khan *et al.* (2005 ▶). For natural products with benzofuran rings, see: Akgul & Anil (2003 ▶); Soekamto *et al.* (2003 ▶). For our previous structural studies of related 3-methyl­sulfinyl-2-phenyl-1-benzofuran derivatives, see: Choi *et al.* (2007 ▶, 2008 ▶).
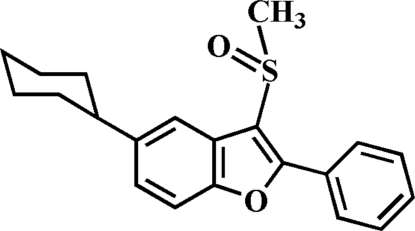


## Experimental

### 

#### Crystal data

C_21_H_22_O_2_S
*M* *_r_* = 338.45
Monoclinic, 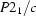

*a* = 9.9864 (2) Å
*b* = 17.1899 (3) Å
*c* = 11.0792 (2) Å
β = 113.540 (1)°
*V* = 1743.64 (6) Å^3^
*Z* = 4
Mo *K*α radiation
μ = 0.20 mm^−1^
*T* = 173 K
0.30 × 0.23 × 0.15 mm

#### Data collection

Bruker SMART APEXII CCD diffractometer
Absorption correction: multi-scan (*SADABS*; Bruker, 2009 ▶) *T*_min_ = 0.944, *T*_max_ = 0.972
16429 measured reflections
4009 independent reflections
3171 reflections with *I* > 2σ(*I*)
*R*_int_ = 0.036

#### Refinement

*R*[*F* ^2^ > 2σ(*F* ^2^)] = 0.040
*wR*(*F* ^2^) = 0.103
*S* = 1.04
4009 reflections
218 parameters
H-atom parameters constrained
Δρ_max_ = 0.25 e Å^−3^
Δρ_min_ = −0.40 e Å^−3^

### 

Data collection: *APEX2* (Bruker, 2009 ▶); cell refinement: *SAINT* (Bruker, 2009 ▶); data reduction: *SAINT*; program(s) used to solve structure: *SHELXS97* (Sheldrick, 2008 ▶); program(s) used to refine structure: *SHELXL97* (Sheldrick, 2008 ▶); molecular graphics: *ORTEP-3* (Farrugia, 1997 ▶) and *DIAMOND* (Brandenburg, 1998 ▶); software used to prepare material for publication: *SHELXL97*.

## Supplementary Material

Crystal structure: contains datablocks global, I. DOI: 10.1107/S1600536810054358/xu5130sup1.cif

Structure factors: contains datablocks I. DOI: 10.1107/S1600536810054358/xu5130Isup2.hkl

Additional supplementary materials:  crystallographic information; 3D view; checkCIF report

## Figures and Tables

**Table 1:** Hydrogen-bond geometry (Å, °) *Cg* is the centroid of the C2–C7 benzene ring.

*D*—H⋯*A*	*D*—H	H⋯*A*	*D*⋯*A*	*D*—H⋯*A*
C5—H5⋯O2^i^	0.95	2.50	3.429 (2)	167
C21—H21*B*⋯O2^ii^	0.98	2.33	3.290 (2)	165
C19—H19⋯*Cg*^ii^	0.95	2.59	3.392 (2)	142

Symmetry codes: (i) 
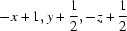
; (ii) 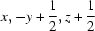.

**Fig. 1.**
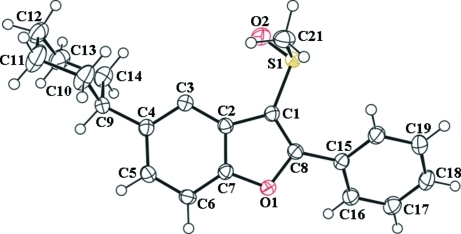
The molecular structure of the title compound with the atom numbering scheme. Displacement ellipsoids are drawn at the 50% probability level. H atoms are presented as a small spheres of arbitrary radius.

**Fig. 2.**
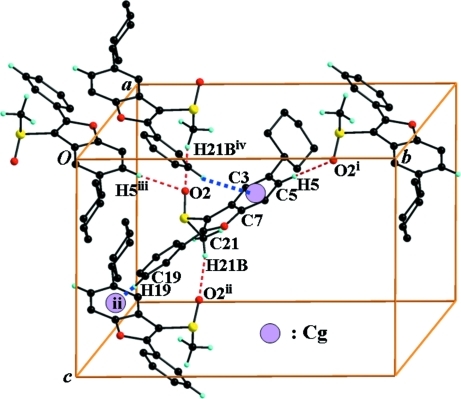
A view of the C–H···O and C–H···π interactions (dotted lines) in the crystal structure of the title compound. Cg denotes the centroid of the C2-C7 benzene ring. [Symmetry codes: (i) - x + 1, y + 1/2, - z + 1/2; (ii) x, - y + 1/2, z + 1/2 ; (iii) - x + 1, y - 1/2, - z + 1/2; (v) x, - y + 1/2, z - 1/2.]

## supplementary crystallographic information

### Comment

Many compounds possessing a benzofuran ring system have received much attention in view of their important pharmacological properties such as antifungal, antimicrobial, antitumor and antiviral activities (Aslam *et al.*, 2006; Galal *et al.*, 2009; Khan *et al.*, 2005). These compounds widely occur in nature (Akgul & Anil, 2003; Soekamto *et al.*, 2003). As part of our ongoing program of the substituent effect on the solid state structures of 3-methylsulfinyl-2-phenyl-1-benzofuran analogues (Choi *et al.*, 2007, 2008), we report herein on the crystal structure of the title compound.

In the title molecule (Fig. 1), the benzofuran unit is essentially planar, with a mean deviation of 0.025 (1) Å from the least-squares plane defined by the nine constituent atoms. The cyclohexyl ring is in the chair form. The phenyl ring makes a dihedral angle of33.38 (5)°  with the mean plane of the benzofuran ring. The crystal packing (Fig. 2) is stabilized by weak intermolecular C—H···O hydrogen bonds; the first one between a benzene H atom and the oxygen of the S═O unit (Table 1; C5—H5···O2^i^), and the second one between a methyl H atom and the oxygen of the S═O unit (Table 1; C21—H21B···O2^ii^). The crystal packing (Fig. 2) is further stabilized by an intermolecular C–H···π interaction between the phenyl H atom and the benzene ring (Table 1; C19—H19···Cg^ii^, Cg is the centroid of the C2–C7 benzene ring).

### Experimental

77% 3-Chloroperoxybenzoic acid (269 mg, 1.2 mmol) was added in small portions to a stirred solution of 5-cyclohexyl-3-methylsulfanyl-2-phenyl-1-benzofuran (386 mg, 1.2 mmol) in dichloromethane (40 mL) at 273 K. After being stirred at room temperature for 4 h, the mixture was washed with saturated sodium bicarbonate solution, and the organic layer was separated, dried over magnesium sulfate, filtered and concentrated at reduced pressure. The residue was purified by column chromatography (hexane-ethyl acetate, 2:1 v/v) to afford the title compound as a colorless solid [yield 71%, m.p. 445-446 K;  *R*_f_ = 0.55 (hexane-ethyl acetate, 2:1 v/v)]. Single crystals suitable for X-ray diffraction were prepared by slow evaporation of a solution of the title compound in acetone at room temperature.

### Refinement

All H atoms were positioned geometrically and refined using a riding model, with C–H = 0.95 Å for aryl, 1.00 Å for methine, 0.99 Å for methylene and 0.98 Å for methyl H atoms, respectively. *U*_iso_(H) = 1.2*U*_eq_(C) for aryl, methine and methylene, and 1.5*U*_eq_(C) for methyl H atoms.

### Crystal data

**Table:**

C_21_H_22_O_2_S	*F*(000) = 720
*M**_r_* = 338.45	*D*_x_ = 1.289 Mg m^−^^3^
Monoclinic, *P*2_1_/*c*	Mo *K*α radiation, λ = 0.71073 Å
Hall symbol: -P 2ybc	Cell parameters from 5143 reflections
*a* = 9.9864 (2) Å	θ = 2.3–27.2°
*b* = 17.1899 (3) Å	µ = 0.20 mm^−^^1^
*c* = 11.0792 (2) Å	*T* = 173 K
β = 113.540 (1)°	Block, colourless
*V* = 1743.64 (6) Å^3^	0.30 × 0.23 × 0.15 mm
*Z* = 4	

### Data collection

**Table:**

Bruker SMART APEXII CCD diffractometer	4009 independent reflections
Radiation source: rotating anode	3171 reflections with *I* > 2σ(*I*)
graphite multilayer	*R*_int_ = 0.036
Detector resolution: 10.0 pixels mm^-1^	θ_max_ = 27.5°, θ_min_ = 2.2°
φ and ω scans	*h* = −11→12
Absorption correction: multi-scan (*SADABS*; Bruker, 2009)	*k* = −20→22
*T*_min_ = 0.944, *T*_max_ = 0.972	*l* = −14→14
16429 measured reflections	

### Refinement

**Table:**

Refinement on *F*^2^	Primary atom site location: structure-invariant direct methods
Least-squares matrix: full	Secondary atom site location: difference Fourier map
*R*[*F*^2^ > 2σ(*F*^2^)] = 0.040	Hydrogen site location: difference Fourier map
*wR*(*F*^2^) = 0.103	H-atom parameters constrained
*S* = 1.04	*w* = 1/[σ^2^(*F*_o_^2^) + (0.0471*P*)^2^ + 0.5848*P*] where *P* = (*F*_o_^2^ + 2*F*_c_^2^)/3
4009 reflections	(Δ/σ)_max_ = 0.001
218 parameters	Δρ_max_ = 0.25 e Å^−^^3^
0 restraints	Δρ_min_ = −0.40 e Å^−^^3^

### Special details

**Table:**

Geometry. All esds (except the esd in the dihedral angle between two l.s. planes) are estimated using the full covariance matrix. The cell esds are taken into account individually in the estimation of esds in distances, angles and torsion angles; correlations between esds in cell parameters are only used when they are defined by crystal symmetry. An approximate (isotropic) treatment of cell esds is used for estimating esds involving l.s. planes.
Refinement. Refinement of F^2^ against ALL reflections. The weighted R-factor wR and goodness of fit S are based on F^2^, conventional R-factors R are based on F, with F set to zero for negative F^2^. The threshold expression of F^2^ > 2sigma(F^2^) is used only for calculating R-factors(gt) etc. and is not relevant to the choice of reflections for refinement. R-factors based on F^2^ are statistically about twice as large as those based on F, and R- factors based on ALL data will be even larger.

### Fractional atomic coordinates and isotropic or equivalent isotropic displacement parameters (Å^2^)

**Table:**

	*x*	*y*	*z*	*U*_iso_*/*U*_eq_	
S1	0.53993 (4)	0.23636 (2)	0.45818 (4)	0.02703 (12)	
O1	0.26640 (10)	0.41250 (6)	0.40179 (10)	0.0251 (2)	
O2	0.59564 (12)	0.22776 (7)	0.35222 (11)	0.0338 (3)	
C1	0.44415 (16)	0.32524 (8)	0.42968 (14)	0.0232 (3)	
C2	0.48052 (15)	0.39562 (8)	0.37720 (13)	0.0228 (3)	
C3	0.58967 (16)	0.41863 (9)	0.33612 (15)	0.0264 (3)	
H3	0.6672	0.3843	0.3439	0.032*	
C4	0.58291 (16)	0.49283 (9)	0.28359 (15)	0.0263 (3)	
C5	0.46893 (16)	0.54323 (9)	0.27576 (15)	0.0272 (3)	
H5	0.4672	0.5942	0.2421	0.033*	
C6	0.35948 (16)	0.52148 (9)	0.31502 (14)	0.0262 (3)	
H6	0.2826	0.5559	0.3090	0.031*	
C7	0.36791 (15)	0.44686 (8)	0.36363 (14)	0.0229 (3)	
C8	0.31508 (16)	0.33785 (8)	0.44035 (14)	0.0235 (3)	
C9	0.69304 (18)	0.51956 (9)	0.23033 (16)	0.0305 (3)	
H9	0.6597	0.5715	0.1882	0.037*	
C10	0.84676 (19)	0.53061 (12)	0.33740 (18)	0.0432 (5)	
H10A	0.8423	0.5662	0.4060	0.052*	
H10B	0.8848	0.4799	0.3794	0.052*	
C11	0.9503 (2)	0.56417 (13)	0.2798 (2)	0.0543 (6)	
H11A	0.9179	0.6173	0.2462	0.065*	
H11B	1.0498	0.5680	0.3501	0.065*	
C12	0.9547 (2)	0.51392 (12)	0.16882 (18)	0.0424 (4)	
H12A	1.0142	0.5402	0.1277	0.051*	
H12B	1.0021	0.4637	0.2053	0.051*	
C13	0.8030 (2)	0.49870 (13)	0.06471 (18)	0.0437 (4)	
H13A	0.8096	0.4616	−0.0010	0.052*	
H13B	0.7615	0.5479	0.0185	0.052*	
C14	0.70227 (19)	0.46570 (11)	0.12466 (16)	0.0369 (4)	
H14A	0.7390	0.4142	0.1639	0.044*	
H14B	0.6034	0.4584	0.0546	0.044*	
C15	0.21597 (16)	0.28936 (9)	0.47555 (14)	0.0242 (3)	
C16	0.06542 (16)	0.30137 (10)	0.41243 (15)	0.0307 (4)	
H16	0.0290	0.3419	0.3493	0.037*	
C17	−0.03071 (18)	0.25502 (10)	0.44095 (17)	0.0347 (4)	
H17	−0.1329	0.2638	0.3980	0.042*	
C18	0.02156 (18)	0.19558 (10)	0.53227 (17)	0.0348 (4)	
H18	−0.0447	0.1632	0.5511	0.042*	
C19	0.17003 (19)	0.18356 (10)	0.59575 (16)	0.0343 (4)	
H19	0.2056	0.1427	0.6582	0.041*	
C20	0.26770 (17)	0.23051 (10)	0.56922 (15)	0.0300 (3)	
H20	0.3698	0.2226	0.6149	0.036*	
C21	0.69381 (18)	0.26628 (10)	0.60208 (16)	0.0351 (4)	
H21A	0.7661	0.2243	0.6303	0.053*	
H21B	0.6617	0.2782	0.6729	0.053*	
H21C	0.7378	0.3128	0.5818	0.053*	

### Atomic displacement parameters (Å^2^)

**Table:**

	*U*^11^	*U*^22^	*U*^33^	*U*^12^	*U*^13^	*U*^23^
S1	0.0289 (2)	0.0206 (2)	0.0359 (2)	0.00143 (15)	0.01754 (16)	0.00194 (15)
O1	0.0231 (5)	0.0232 (5)	0.0314 (5)	0.0003 (4)	0.0135 (4)	0.0002 (4)
O2	0.0381 (6)	0.0321 (6)	0.0374 (6)	0.0024 (5)	0.0215 (5)	−0.0055 (5)
C1	0.0230 (7)	0.0216 (7)	0.0257 (7)	−0.0010 (6)	0.0106 (6)	−0.0007 (6)
C2	0.0231 (7)	0.0205 (7)	0.0244 (7)	−0.0007 (6)	0.0090 (6)	−0.0005 (6)
C3	0.0236 (7)	0.0244 (8)	0.0331 (8)	0.0022 (6)	0.0132 (6)	0.0009 (6)
C4	0.0258 (8)	0.0245 (8)	0.0302 (7)	−0.0031 (6)	0.0129 (6)	−0.0007 (6)
C5	0.0288 (8)	0.0212 (7)	0.0314 (8)	−0.0012 (6)	0.0120 (6)	0.0010 (6)
C6	0.0252 (7)	0.0221 (7)	0.0313 (8)	0.0035 (6)	0.0111 (6)	−0.0005 (6)
C7	0.0221 (7)	0.0232 (7)	0.0244 (7)	−0.0023 (6)	0.0103 (6)	−0.0024 (6)
C8	0.0240 (7)	0.0224 (7)	0.0231 (7)	0.0000 (6)	0.0085 (6)	−0.0004 (6)
C9	0.0322 (8)	0.0226 (8)	0.0430 (9)	0.0012 (6)	0.0216 (7)	0.0057 (7)
C10	0.0381 (10)	0.0536 (12)	0.0460 (10)	−0.0186 (9)	0.0253 (8)	−0.0201 (9)
C11	0.0453 (11)	0.0603 (13)	0.0703 (14)	−0.0240 (10)	0.0368 (10)	−0.0203 (11)
C12	0.0360 (9)	0.0549 (12)	0.0450 (10)	0.0003 (8)	0.0254 (8)	0.0062 (9)
C13	0.0409 (10)	0.0590 (12)	0.0369 (9)	0.0063 (9)	0.0214 (8)	0.0103 (9)
C14	0.0333 (9)	0.0461 (11)	0.0309 (8)	−0.0042 (8)	0.0125 (7)	−0.0002 (7)
C15	0.0249 (7)	0.0258 (7)	0.0244 (7)	−0.0020 (6)	0.0124 (6)	−0.0022 (6)
C16	0.0261 (8)	0.0333 (9)	0.0327 (8)	0.0013 (7)	0.0117 (6)	0.0057 (7)
C17	0.0240 (8)	0.0410 (10)	0.0392 (9)	−0.0017 (7)	0.0127 (7)	0.0028 (7)
C18	0.0339 (9)	0.0363 (9)	0.0401 (9)	−0.0081 (7)	0.0210 (7)	0.0005 (7)
C19	0.0372 (9)	0.0352 (9)	0.0342 (8)	0.0018 (7)	0.0182 (7)	0.0090 (7)
C20	0.0256 (8)	0.0364 (9)	0.0289 (8)	0.0016 (7)	0.0118 (6)	0.0054 (7)
C21	0.0333 (9)	0.0401 (10)	0.0312 (8)	0.0106 (7)	0.0121 (7)	0.0043 (7)

### Geometric parameters (Å, °)

**Table:**

S1—O2	1.4938 (12)	C11—H11A	0.9900
S1—C1	1.7628 (15)	C11—H11B	0.9900
S1—C21	1.7919 (17)	C12—C13	1.516 (3)
O1—C7	1.3775 (17)	C12—H12A	0.9900
O1—C8	1.3783 (17)	C12—H12B	0.9900
C1—C8	1.358 (2)	C13—C14	1.519 (2)
C1—C2	1.450 (2)	C13—H13A	0.9900
C2—C7	1.389 (2)	C13—H13B	0.9900
C2—C3	1.396 (2)	C14—H14A	0.9900
C3—C4	1.392 (2)	C14—H14B	0.9900
C3—H3	0.9500	C15—C20	1.392 (2)
C4—C5	1.405 (2)	C15—C16	1.397 (2)
C4—C9	1.512 (2)	C16—C17	1.378 (2)
C5—C6	1.380 (2)	C16—H16	0.9500
C5—H5	0.9500	C17—C18	1.385 (2)
C6—C7	1.381 (2)	C17—H17	0.9500
C6—H6	0.9500	C18—C19	1.379 (2)
C8—C15	1.461 (2)	C18—H18	0.9500
C9—C14	1.524 (2)	C19—C20	1.385 (2)
C9—C10	1.531 (2)	C19—H19	0.9500
C9—H9	1.0000	C20—H20	0.9500
C10—C11	1.528 (2)	C21—H21A	0.9800
C10—H10A	0.9900	C21—H21B	0.9800
C10—H10B	0.9900	C21—H21C	0.9800
C11—C12	1.517 (3)		
			
O2—S1—C1	106.90 (7)	C10—C11—H11B	109.3
O2—S1—C21	105.75 (7)	H11A—C11—H11B	108.0
C1—S1—C21	96.99 (7)	C13—C12—C11	111.81 (16)
C7—O1—C8	106.42 (11)	C13—C12—H12A	109.3
C8—C1—C2	107.47 (13)	C11—C12—H12A	109.3
C8—C1—S1	126.07 (11)	C13—C12—H12B	109.3
C2—C1—S1	126.16 (11)	C11—C12—H12B	109.3
C7—C2—C3	119.28 (13)	H12A—C12—H12B	107.9
C7—C2—C1	104.54 (13)	C12—C13—C14	111.50 (14)
C3—C2—C1	136.10 (14)	C12—C13—H13A	109.3
C4—C3—C2	118.83 (14)	C14—C13—H13A	109.3
C4—C3—H3	120.6	C12—C13—H13B	109.3
C2—C3—H3	120.6	C14—C13—H13B	109.3
C3—C4—C5	119.56 (14)	H13A—C13—H13B	108.0
C3—C4—C9	121.33 (14)	C13—C14—C9	111.27 (15)
C5—C4—C9	119.07 (13)	C13—C14—H14A	109.4
C6—C5—C4	122.52 (14)	C9—C14—H14A	109.4
C6—C5—H5	118.7	C13—C14—H14B	109.4
C4—C5—H5	118.7	C9—C14—H14B	109.4
C5—C6—C7	116.25 (14)	H14A—C14—H14B	108.0
C5—C6—H6	121.9	C20—C15—C16	119.02 (14)
C7—C6—H6	121.9	C20—C15—C8	121.61 (13)
O1—C7—C6	125.48 (13)	C16—C15—C8	119.36 (13)
O1—C7—C2	110.99 (12)	C17—C16—C15	120.58 (15)
C6—C7—C2	123.51 (14)	C17—C16—H16	119.7
C1—C8—O1	110.56 (12)	C15—C16—H16	119.7
C1—C8—C15	134.42 (14)	C16—C17—C18	120.02 (15)
O1—C8—C15	114.91 (12)	C16—C17—H17	120.0
C4—C9—C14	113.06 (13)	C18—C17—H17	120.0
C4—C9—C10	113.30 (13)	C19—C18—C17	119.86 (15)
C14—C9—C10	108.72 (14)	C19—C18—H18	120.1
C4—C9—H9	107.1	C17—C18—H18	120.1
C14—C9—H9	107.1	C18—C19—C20	120.60 (15)
C10—C9—H9	107.1	C18—C19—H19	119.7
C11—C10—C9	111.05 (16)	C20—C19—H19	119.7
C11—C10—H10A	109.4	C19—C20—C15	119.90 (14)
C9—C10—H10A	109.4	C19—C20—H20	120.1
C11—C10—H10B	109.4	C15—C20—H20	120.1
C9—C10—H10B	109.4	S1—C21—H21A	109.5
H10A—C10—H10B	108.0	S1—C21—H21B	109.5
C12—C11—C10	111.51 (16)	H21A—C21—H21B	109.5
C12—C11—H11A	109.3	S1—C21—H21C	109.5
C10—C11—H11A	109.3	H21A—C21—H21C	109.5
C12—C11—H11B	109.3	H21B—C21—H21C	109.5
			
O2—S1—C1—C8	138.30 (13)	C7—O1—C8—C1	1.09 (15)
C21—S1—C1—C8	−112.85 (14)	C7—O1—C8—C15	−175.64 (12)
O2—S1—C1—C2	−34.61 (14)	C3—C4—C9—C14	55.4 (2)
C21—S1—C1—C2	74.24 (14)	C5—C4—C9—C14	−122.56 (16)
C8—C1—C2—C7	1.53 (16)	C3—C4—C9—C10	−68.92 (19)
S1—C1—C2—C7	175.53 (11)	C5—C4—C9—C10	113.16 (17)
C8—C1—C2—C3	−175.00 (16)	C4—C9—C10—C11	−175.16 (15)
S1—C1—C2—C3	−1.0 (3)	C14—C9—C10—C11	58.2 (2)
C7—C2—C3—C4	0.5 (2)	C9—C10—C11—C12	−56.0 (2)
C1—C2—C3—C4	176.69 (15)	C10—C11—C12—C13	52.9 (2)
C2—C3—C4—C5	1.4 (2)	C11—C12—C13—C14	−53.3 (2)
C2—C3—C4—C9	−176.53 (14)	C12—C13—C14—C9	56.9 (2)
C3—C4—C5—C6	−1.8 (2)	C4—C9—C14—C13	174.51 (14)
C9—C4—C5—C6	176.11 (14)	C10—C9—C14—C13	−58.75 (18)
C4—C5—C6—C7	0.3 (2)	C1—C8—C15—C20	34.6 (3)
C8—O1—C7—C6	178.45 (14)	O1—C8—C15—C20	−149.65 (14)
C8—O1—C7—C2	−0.06 (15)	C1—C8—C15—C16	−144.36 (17)
C5—C6—C7—O1	−176.58 (13)	O1—C8—C15—C16	31.35 (19)
C5—C6—C7—C2	1.7 (2)	C20—C15—C16—C17	−0.9 (2)
C3—C2—C7—O1	176.35 (12)	C8—C15—C16—C17	178.11 (15)
C1—C2—C7—O1	−0.90 (15)	C15—C16—C17—C18	−0.4 (3)
C3—C2—C7—C6	−2.2 (2)	C16—C17—C18—C19	0.8 (3)
C1—C2—C7—C6	−179.44 (13)	C17—C18—C19—C20	0.1 (3)
C2—C1—C8—O1	−1.65 (16)	C18—C19—C20—C15	−1.4 (3)
S1—C1—C8—O1	−175.65 (10)	C16—C15—C20—C19	1.8 (2)
C2—C1—C8—C15	174.19 (15)	C8—C15—C20—C19	−177.17 (15)
S1—C1—C8—C15	0.2 (2)		

### Hydrogen-bond geometry (Å, °)

**Table:**

Cg is the centroid of the C2–C7 benzene ring.

**Table:**

*D*—H···*A*	*D*—H	H···*A*	*D*···*A*	*D*—H···*A*
C5—H5···O2^i^	0.95	2.50	3.429 (2)	167
C21—H21B···O2^ii^	0.98	2.33	3.290 (2)	165
C19—H19···Cg^ii^	0.95	2.59	3.392 (2)	142

Symmetry codes: (i) −*x*+1, *y*+1/2, −*z*+1/2; (ii) *x*, −*y*+1/2, *z*+1/2.
